# Mass Spectrometry-Based Metabolomic and Proteomic Strategies in Organic Acidemias

**DOI:** 10.1155/2016/9210408

**Published:** 2016-06-14

**Authors:** Esther Imperlini, Lucia Santorelli, Stefania Orrù, Emanuela Scolamiero, Margherita Ruoppolo, Marianna Caterino

**Affiliations:** ^1^IRCCS SDN, 80143 Naples, Italy; ^2^CEINGE Biotecnologie Avanzate s.c.a.r.l., 80145 Naples, Italy; ^3^Dipartimento di Scienze Motorie e del Benessere, Università di Napoli “Parthenope”, 80133 Naples, Italy; ^4^Dipartimento di Medicina Molecolare e Biotecnologie Mediche, Università degli Studi di Napoli “Federico II”, 80121 Naples, Italy; ^5^Associazione Culturale DiSciMuS RCF, Casoria, 80026 Naples, Italy

## Abstract

Organic acidemias (OAs) are inherited metabolic disorders caused by deficiency of enzymatic activities in the catabolism of amino acids, carbohydrates, or lipids. These disorders result in the accumulation of mono-, di-, or tricarboxylic acids, generally referred to as organic acids. The OA outcomes can involve different organs and/or systems. Some OA disorders are easily managed if promptly diagnosed and treated, whereas, in others cases, such as propionate metabolism-related OAs (propionic acidemia, PA; methylmalonic acidemia, MMA), neither diet, vitamin therapy, nor liver transplantation appears to prevent multiorgan impairment. Here, we review the recent developments in dissecting molecular bases of OAs by using integration of mass spectrometry- (MS-) based metabolomic and proteomic strategies. MS-based techniques have facilitated the rapid and economical evaluation of a broad spectrum of metabolites in various body fluids, also collected in small samples, like dried blood spots. This approach has enabled the timely diagnosis of OAs, thereby facilitating early therapeutic intervention. Besides providing an overview of MS-based approaches most frequently used to study the molecular mechanisms underlying OA pathophysiology, we discuss the principal challenges of metabolomic and proteomic applications to OAs.

## 1. Introduction

The term “inborn errors of metabolism” (IEMs) was coined by Garrod in 1908 to describe genetically determined conditions, such as alkaptonuria, albinism, pentosuria, and cystinuria [1]. Nowadays IEMs represent genetic disorders that are caused by alterations of a specific enzymatic reaction. Although individually rare, IEMs collectively account for a significant proportion of genetic illnesses, particularly in children. IEMs can be pleiotropic and can involve virtually any organ or system. Initial clinical presentation can occur any time from prenatal development through adulthood, and specific environmental triggers are crucial to determine an individual patient phenotype [2].

One of the primary challenges presented by IEMs is their extreme diversity, which has always made them difficult to classify. Currently, IEMs are categorized according to the affected organ (as in “neurological” or “hepatic” diseases) or to the affected organelle (e.g., “mitochondrial,” “peroxisomal,” or “lysosomal” disorders) or to the age of presentation (neonatal or adult-onset IEM). Because each of these approaches is informative, no single, universal classification system exists [3].

The genetic basis of IEMs is extremely heterogeneous and can involve any type of genetic defect: one or more point mutations, deletions or insertions, or genomic rearrangements (see Supplementary Table 1 in Supplementary Material available online at http://dx.doi.org/10.1155/2016/9210408). Mutations can occur in coding or regulatory sequences, and mutations occurring in different genes can affect the same pathway. The pathogenesis of IEM can generally be attributed to the loss- or gain-of-function of mutant proteins (usually enzymes or transporters), which in turn are able to alter a specific metabolite flux within a determined cell pathway.

The biological effects of IEM mutations can be mediated by four main processes: (a) direct toxicity of accumulating upstream metabolites; (b) deficiency of downstream metabolites; (c) feedback inhibition or activation by the metabolite on the same or different pathway; and (d) diversion of metabolic flux to secondary pathways [3].

Organic acidemias or organic acidurias (OAs) are inherited metabolic disorders caused by deficiency of enzymatic activities in the catabolism of amino acids, carbohydrates, or lipids [4, 5]. These disorders result in the accumulation of mono-, di-, or tricarboxylic acids, generally referred to as organic acids. They include maple syrup urine disease (MSUD), propionic acidemia (PA), methylmalonic acidemia (MMA), isovaleric acidemia (IVA), 3-hydroxy-3-methylglutaryl-CoA (HMG-CoA) lyase deficiency, beta ketothiolase deficiency (*β*-KTD), and glutaric acidemia type I (GA I). Among OA common clinical symptoms there are a toxic encephalopathy, with seizures, hypotonia, lethargy, coma, vomiting, respiratory distress, and poor feeding in the neonatal period. For specific OAs, clinical features may occur in the older child or adolescence determining progressive neurologic deterioration, loss of intellectual function, ataxia, Reye syndrome, recurrent ketoacidosis, or psychiatric symptoms. The OA outcomes can involve different organs and/or systems; the multisystemic nature of the different OAs is showed in Table 1. Therapy, often imperfect at best, includes dietary manipulation to reduce the enzyme substrate and/or administration of appropriate vitamins to increase the residual activity of the mutant enzyme and/or increase of the excretion of potentially toxic metabolites by providing glycine and/or L-carnitine and, in some cases, by supplying enzyme in the form of liver transplantation [6–8].

Some OA disorders are easily managed if promptly diagnosed and treated, but, in others cases, functional deterioration of brain and other organs with high energy demands are quite common [9]. In this regard, it has been suggested that the formation of the toxic metabolites may occur within the brain [9]. Moreover, the energetic functional impairment of other organs or systems in OAs may be related to a preferential accumulation of mitochondrial intermediates within this same organelle, before leaving the cell [9]. This is particularly true for OAs related to propionate metabolism such as MMA and PA, in which neither diet, vitamin therapy, nor liver transplantation appears to prevent neurologic complications such as leukoencephalopathy and basal ganglia atrophy [9].

PA is caused by mutations in either the propionyl-CoA carboxylase alpha (PCCA) or beta (PCCB) genes, which encode for the mitochondrial enzyme PCC. Deficiency of PCC activity leads to accumulation and excretion of propionate, 3-hydroxypropionate, methylcitrate, and propionylglycine, as well as ammonia and lactate, especially during metabolic crises [10]. Abnormal mitochondrial structure has been demonstrated in skeletal muscle, heart, and liver from PA patients, together with severe impairment of oxidative phosphorylation [11].

Two enzymatic phenotypes of apoenzyme deficiency are recognized for MMA, both involving the methylmalonyl-CoA mutase (MUT): fibroblasts from* mut* ^0^ patients have no detectable or residual enzyme activity while those from* mut* ^−^ patients show a markedly reduced enzyme activity [12]. MUT converts L-methylmalonyl-CoA into succinyl-CoA, a Krebs cycle intermediate. A block at this enzymatic step results in elevated plasma levels of methylmalonic acid as well in the accumulation of other propionyl-CoA-derived metabolites, such as 2-methylcitrate [10]. Patients, carrying mutations in the* MUT *gene, typically have severe disease and demonstrate poor outcomes, with early mortality and substantial lifelong morbidity [13–15].

MMA can also be triggered by an aberrant intracellular metabolism of MUT cofactor, vitamin B12 (also referred to as cobalamin, Cbl). In particular seven enzymes are responsible for the transport, processing, and delivery of the appropriate Cbl form via a mitochondrion-targeted route to MUT [16, 17]. Mutations in any of them define three broad disease phenotypes: isolated MMA, combined MMA, and homocystinuria [18]. These disorders are classified—either Cbl A, B, C, D, E, F, or G—and are also clinically and biochemically heterogeneous [19, 20]. Cobalamin C (Cbl-C) defect is the most common inborn error of cobalamin metabolism and causes combined MMA with homocystinuria. Despite pharmacological treatment with OH-Cbl, betaine, folic acid, and carnitine, the long-term outcome is in most cases unsatisfactory with progression of neurological and visual impairment, mainly expressed in form of retinal degeneration and/or maculopathy.

Here, we review the latest results in dissecting molecular bases of OAs by using integration of mass spectrometry-based metabolomic and proteomic strategies. Generally, measurements of metabolites in various body fluids are the current tools for diagnosis. Mass spectrometry (MS) has facilitated the rapid and economical evaluation of a broad spectrum of metabolites from small samples, including dried blood spots. This approach has enabled the timely diagnosis of OAs, thereby facilitating early institution of therapy.

## 2. Metabolomic Strategies and Applications in Organic Acidemias

The term “metabolome” was introduced by Oliver et al. to indicate “the holistic quantitative set of low molecular-weight compounds (<1000 Da)” [21] in the 1998. However the study of metabolome has more ancient origin. The word metabolism originates from Greek language and denotes “change.” Ancient cultures used urine color, taste, and smell for diabetes diagnosis, as their change is related to different metabolite levels [22]. In 1950s Williams reported the presence of a interindividual variable but intraindividual constant metabolic pattern [23]. Despite the fact that the first experimental evidences supporting the metabolic pattern analysis might have clinical utility, no further investigation was performed until the 70s, when a boost came by the rapid increase of technology in gas chromatography (GC), liquid chromatography (LC), and MS field. In the 1970s two key papers published by E. C. Horning and M. G. Horning and by Pauling et al. stated that the functional status of a complex biological system resides in the quantitative and qualitative pattern of metabolites in body fluids [24, 25]. Nicholson et al. introduced the term metabonomics to indicate the profiling of whole metabolic composition in a living system with simultaneous determination of their changes resulting from multiple environmental conditions as well as genetic background [26]. The term metabolomics was used, firstly, by Fiehn in 2002, indicating the comprehensive and quantitative analysis of all metabolites in a system [27]. Nowadays the terms metabonomics and metabolomics are used interchangeably and provide a generalised outline to characterize metabolite profiles of complex biochemical mixtures. In this regard, metabolomics enables the identification of the downstream effects caused by the action of genes and proteins, thus allowing defining what is currently taking place. Hence, metabolomics not only enables the identification of disease biomarkers in the form of endogenous metabolites (gene-derived metabolites) and exogenous metabolites (environmentally derived metabolites) but also provides unique insights into the fundamental causes of disease [28].

### 2.1. Metabolomic Workflow

An appropriate study design is crucial to ensure the correct data interpretation of metabolic experiments. The inclusion of a sufficient number of subjects in each group, the sample collection, the choice of technological platforms, the processing of generated data, and the application of various bioinformatic methods are all factors that maximize analytical power of metabolomic approach aimed at identifying compounds and pathways of interest [29].

**Figure 1:**
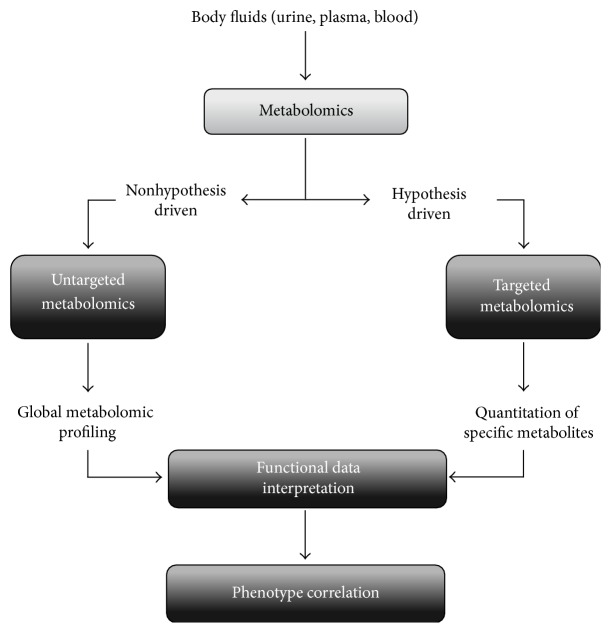
Schematic view of metabolomic methods. Samples deriving from body fluids (i.e., urine, plasma, and blood) are source for metabolomics. Two different strategies can be adopted. Targeted metabolomics allows the quantitation of a limited number of metabolites based on an a priori hypothesis. Untargeted metabolomics allows the determination of all the metabolites detectable in biofluids, without an a priori hypothesis. Biological interpretation of qualitative and quantitative alterations of metabolomics dataset correlates the metabolite patterns to biological pathways and cellular processes.

Two different strategies, named targeted and untargeted metabolomics, can be adopted, for this purpose (Figure 1). Targeted metabolomics is a specific and quantitative approach that enables the evaluation of a limited number of metabolites based on an a priori hypothesis [30].

On the other hand, untargeted metabolomics is a nonspecific approach whose main aim is to determinate the whole set of metabolites detectable in a fluid or tissue, thus providing a functional fingerprint of the pathophysiological state of the body.

Whereas metabolic targeted and targeted procedures indicate what is happening at a biochemical level, the success of metabolomics in biomarker translation, with respect to other omics techniques, resides in the robustness of the adopted protocols and instrumentations, in the highly quantitative aspect, easily adapted to new assays and already located in many clinical testing laboratories.

Many successful studies have been conducted using urine, plasma, or serum samples. Urine metabolome better reflects kidney pathophysiological changes, while metabolome in whole blood, plasma, and serum is more related to systemic changes. The advantage of metabolomic analysis is to use noninvasive or minimally invasive sample collection procedure. Urine, in fact, easily and noninvasively collected, represents an “open system” that includes the intermediate metabolites, thus reflecting specific metabolic processes [31]. Dried blood spot samples, collected on a suitable inert paper matrix, are also a convenient method for metabolic profiling, thus allowing an easier sample collection, storage and transport [32]. Interestingly, dried blood spot sample does not require a number of preanalytical steps (sample preparation, storage, freezing, and transportation on dry ice), thus considerably simplifying the experimental design [33]. Moreover, it has been reported that the metabolite profiles from blood spots are similar to those obtained from more abundant protein-depleted plasma [34, 35]. However sample collection, storage, and pretreatment could have a negative impact on the outcome of the analysis resulting in incorrect identification of biomarkers. A standard operating procedures need to be considered for the biofluids used for each metabolomic study.

Due to the huge diversity of chemical structures and the large differences in abundance, there is no single technology available to analyze the entire metabolome. The most appropriate methodology may be selected as a compromise between the chemical selectivity, sensitivity, and speed of the different techniques. In addition to MS, also nuclear magnetic resonance (NMR) is used in order to analyze a large number of metabolites (up to 20–50) simultaneously [17, 18], because it requires minimal sample pretreatment and provides highly reproducible quantitative results. However, as analytical limits the NMR is able to mainly determine the high abundant analytes but not less abundant ones, present in a complex matrix. On the contrary the deep power of MS consist of its easiness to be coupled to separation techniques LC-MS, GC-MS, and LC-MS/MS, thus providing (a) the determination of wide spectrum of compounds with several physicochemical properties and different abundance (picomolar and nanomolar range) and (b) high sensitivity and selectivity [36]. As for LC-MS, combination of two complementary chromatographic separations such as reversed-phase LC (RPLC) and hydrophilic interaction LC (HILIC) is often used to ensure the coverage of a large set of metabolites [37, 38]. Recently, ultrahigh performance LC (UHPLC) and two-dimensional LC techniques are successfully used in metabolomics studies allowing a huge improvement of the chromatographic resolution. Conversely, the use of GC technique is more stringent thus demanding the application of derivatization steps in the sample preparation process for the analysis of polar and nonvolatile compounds. Since each technique provides broad coverage of many classes of organic compounds, it is useful in a metabolomics approach to complement each other.

As for processing of generated data, statistical analysis has a great impact on metabolite identification and quantitation and on the resulting biological interpretation. Two common statistical approaches may be adopted: the unsupervised method such as principal components analysis (PCA) and the supervised method, including partial least squares (PLS). In particular, PCA involves the transformation of the variables into a set of unrelated orthogonal components, with the first and a subsequent component explaining the largest and smaller amount of variance in the data, respectively. PCA helps to identify outliers in metabolomic experiments and also identify other technical issues that could produce variability in the data. PLS models correlate a feature of interest with the entire metabolomic dataset, and hence the components of a PLS model indicate how much the particular metabolite contributes to statistical significance of a specific dataset [39].

In order to obtain biological interpretation of whole metabolomic dataset, it is important to correlate metabolites belonging to the same metabolic pathway or chairing common quantitative changes. Hence, metabolomic data can be clustered into different “network” of metabolic pathways, in which nodes represent experimental and known metabolites [40]. Moreover, the availability of public repositories including metabolites within functional route, for example, Kyoto Encyclopedia of Genes and Genomes (KEGG), also allows performing pathway analysis [41].

Moreover, a proper validation of the obtained results represents an important challenge of metabolomic investigation. Hence, it is pivotal to compare the analytical data between different studies and/or laboratories.

### 2.2. Metabolomic Applications to OAs

In the last decade metabolomic approaches were applied in the investigation of IEMs to better understand the molecular processes underlying their development. The recent literature showed the big potential and impact of these approaches especially for diseases characterized by a-symptomatic development.

An example of diagnosis of pre- and a-symptomatic diseases is represented by the targeted metabolomic approach applied to newborn screening for inherited metabolic disorders. The IEMs including OAs were diagnosed by using tandem mass spectrometry since the 1990s [42, 43]. More than 70 different metabolic diseases can be diagnosed by measuring metabolites extracted from a single dried blood spot (DBS) collected within 48–72 hours of birth. The standard MS newborn screening procedure allows the determination of amino acids and acylcarnitines in semiquantitative mode, by using LC-MS/MS analytical platform.  Table 2 shows the biomarkers (amino acids, amino acid ratios, acylcarnitines, and acylcarnitine ratios) analyzed on DBS by LC-MS/MS which are used in the diagnosis of OAs. As shown, propionylcarnitine increases in several diseases: consequently, a second-tier test performed on the DBS by LC-MS/MS analysis is performed to quantitate methylmalonic acid and propionic acid in order to discriminate between MMA and PA [44]. Urinary organic acids performed by GC-MS or acylglycines measured by LC-MS/MS analysis are then used to confirm the abnormal profile [45, 46].  Table 3 shows organic acids and acylglycine identified in OAs.

Dénes et al. [47] explored a new area in the newborn screening field to improve sensitivity and specificity. The authors demonstrated that the high-resolution mass spectrometry (HR-MS) provides higher metabolome coverage comparable to that of canonical LC-MS/MS approaches. These results can reduce false-positive rate. Suspected positive cases, in fact, require confirmatory testing, which increase analytical burden on the metabolic diagnostic laboratories. Besides the detection of amino acids and acylcarnitines, the novel method was also capable of the semiquantitative determination of highly specific disease markers including various organic acids, acylglycines, or even carbohydrates. Routine application of the method was expected to decrease the number of second samples requested as well as the number of second-tier or confirmatory tests. However the high cost of the instrumentation and the need of high qualified personnel involved in the running of the equipment limit the applicability in routine analysis.

Wojtowicz et al. [48] used a GC × GC-MS analytical platform, whose separation power is elevated, to improve analyte detectability of pathological markers of OAs in urine sample. The procedure consists of the following steps: (a) spectral peaks were chosen according to their ratio signal to noise (*S*/*N* > 200); (b) peaks of interest (metabolic biomarkers of pathology, in this case) are exported to the reference, which is a set of information containing the retention times and mass spectrum of each analyte, among other data; (c) the reference is used to search for each analyte in the unknown sample and for the quantification of positively identified analytes. To create a reference applicable for the diagnoses, markers were imported from different samples where the given metabolite was present. The data processing strategy provided significant time-savings compared to classical manual approach. However this kind of instrumentation is not widely employed in diagnostic laboratories, due to high cost and need of bioinformatic expertise to data interpretation.

Moving to untargeted examples, Wikoff et al. used untargeted LC-MS analysis to simultaneously profile thousands of metabolites on plasma of MMA and PA patients in order not only to characterize the metabolomic pattern of the these two diseases but also to define the specific difference between them [49]. They performed a profiling approach, including new nonlinear time correction, peak-finding, and integration methods to allow semiquantitative comparison between healthy individuals and patient populations. The study included plasma samples from healthy adults with (*n* = 3) and without (*n* = 3) carnitine supplementation, healthy children with (*n* = 3) and without (*n* = 12) carnitine supplementation, children with mut^0^ MMA (*n* = 15), and children with PA (*n* = 9). The authors chose a dual analytical platform: LC-MS to determinate unknown metabolite from methanolic plasma sample extracts and LC-MS/MS to confirm the identified metabolites by their specific mass fragmentation pattern. An intriguing result of this study is the significant increase in *γ*-butyrobetaine concentrations in patients with MMA and PA, which has not been previously reported. *γ*-Butyrobetaine is converted to L-carnitine by *γ*-butyrobetaine hydroxylase, localized in liver, kidney, and brain in humans [50]. Increase of *γ*-butyrobetaine may arise by inhibition of *γ*-butyrobetaine hydroxylase by metabolites accumulating in these defects. Alternatively, propionyl carnitine, which accumulates in PA and MMA, inhibits *γ*-butyrobetaine transport across the plasma membrane in liver [51] and thus plasma concentrations. The authors also suggested that C6:1 (or methyl C5:1) was increased in PA relative to MMA, while isovaleryl carnitine was increased in MMA and PA. These results seem to be promising but should be reinforced by the enrollment of higher number of samples.

Finally, an untargeted metabolomic approach was tested by Miller et al. [52] to study 21 different IEMs, including OAs, using three different but complementary mass spectrometry platforms: (a) GC-MS; (b) LC-MS in positive ion mode; and (c) LC-MS in negative ion mode. The authors emphasized the need to have more accurate and speed diagnosis methods to improve therapeutical action. For all analytic methods, metabolites were identified by matching the ion chromatographic retention index, accurate mass, and mass spectral fragmentation signatures with reference library entries, created from authentic standard metabolites by means of the same analytical procedure [53]. The primary advantage of this kind of approach is the simultaneous detection of more than 400 endogenous analytes. Results reported consistent detection of key biomarkers across many specimens with clear segregation of disease-related analyte levels between unaffected versus affected individuals in nearly all cases tested. Overall this analysis provided excellent coverage of the amino acids and acylcarnitines performed by targeted methods; however a number a relevant plasma compounds like homocysteine and methylmalonic acid were not identified. As the authors stated, the initial proof-of-concept study is encouraging, but further improvements are needed to test reproducibility of this platform across multiple independent analysis.

In this regard, the recent technological advancements in MS may certainly promote the automation of the MS-based metabolomics analysis, thus allowing (a) reducing costs, (b) increasing throughput, (c) ensuring greater reproducibility, substantially cutting down on sample-handling errors, and (d) encouraging a greater focus on the absolute quantification. So the automation of technologies represents a great improvement especially when a high number of metabolomic analyses is required to reduce the number of false positives (normal sample reported as abnormal) and false negative (abnormal sample reported as normal). To date, the most successful example of metabolomic application to OAs is represented by metabolic targeted methods utilized in the newborn screening. Not only does MS-based newborn screening help in diagnosing or even predicting disease, but also the same techniques can also be used to determine the optimal therapy and monitor or customize the therapeutic dose. One of the main reasons for the success and widespread adoption of high-throughput MS-based screening is the very low sample costs.

However, a major limit of the metabolomic strategies is actually the limited number of identified metabolites due to the small metabolite coverage obtained by the so far developed MS profiling methods. It is well known that biological interpretation has to be performed on a high number of metabolites. It is challenging to get a good biological interpretation based on only fragments of the overall picture.

## 3. Proteomics Strategies and Applications in Organic Acidemias

Proteomics has the potential to complement metabolomics and contribute to a better understanding of disease processes. The term “proteome” was first used by Wilkins et al. in 1996 to indicate snapshots of protein composition from a particular tissue or organism, at defined time points and under given physiological (or pathological) conditions [54]. A qualitative proteomic analysis is focused on the study of proteins present in various types of biological materials, in particular to identify their functions, structures, and interaction sites or posttranslational modifications (PTMs) [55, 56]. Towards this aim, a multitude of MS-based proteomic approaches have been developed, and although proteomic studies represent a fruitful field in molecular research, comprehensive quantitative descriptions of biological systems at protein levels are more recent [57]. In fact, the fast-evolving MS techniques, the identification, and quantitation of all of the proteins in a biological system are still an experimental challenge. However, successful impact of proteomics in biomedical science has prompted clinicians to use innovative proteomic technology into clinical research, thus considering its application to the translational medicine. This has indeed triggered the burst of clinical proteomics, thus allowing (a) the unravelling the disease-related molecular mechanisms and (b) the identification of new disease biomarkers to be used in clinical applications for diagnosis, for evaluation of therapy outcomes and for follow-up analyses [58–60]. Clinical proteomics, in fact, enable the quantitative and qualitative profiling of proteins and peptides that are present in clinical specimens like body fluids, cells, and tissues [61, 62].

### 3.1. Proteomic Workflow

**Figure 2:**
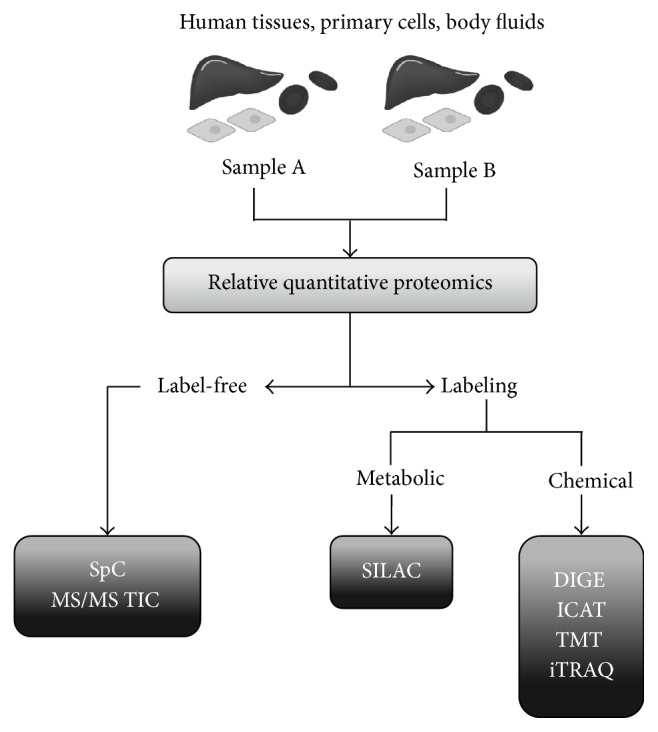
Schematic view of quantitative proteomic methods. Samples deriving from patients (i.e., tissues, cells, and body fluids) are the sources for clinical proteomics. Label-free and labeling proteomic approaches are the two main groups of MS-based strategies aimed at identifying and quantifying differentially expressed proteins between two different samples A and B (i.e., cells or tissues from OA patients versus healthy controls). Label-free methods include SpC and MS/MS TIC approaches. On the other hand, the other methods are based on metabolic labeling, such as SILAC, or chemical such as DIGE, ICAT, TMT, and iTRAQ.

In most quantitative proteomic workflows, MS-based procedures can be grouped in two major approaches: labeling and label-free methods [63, 64] (Figure 2). Among the two approaches, the first provides certainly the most accurate quantitation through chemical (Two-Dimensional Differential In-Gel Electrophoresis, 2D-DIGE; Isotope Coded Affinity Tags, ICAT; Tandem Mass Tags, TMT; Isobaric Tag for Relative and Absolute Quantitation, iTRAQ) and metabolic (Stable Isotope Labeling by Amino Acids in Cell Culture, SILAC) labeling [62–69], whereas label-free methods (Spectral Counting, SpC; MS/MS Total Ion Current, MS^2^ TIC), widely spread for their ease of use, enable relative quantitation over a large dynamic range in comparison to labeling approaches [70–73] (Figure 2).

The classical quantitative proteomic methods utilize dyes coupled to a high-resolution protein separation technique, such as 2D electrophoresis. In particular, the use of fluorescent dyes in 2D-DIGE protocols increases sensitivity, offers a linear dynamic range, and allows both the quantitative comparison of gel-based protein patterns and their identification by MALDI-TOF or by LC-MS/MS techniques [74, 75].

The other accurate quantitative approaches are based on stable isotope labeling; in this case, quantitation is achieved by comparing mass spectrometric signal intensities between corresponding labeled and unlabeled peptides. Isotope labels can be introduced chemically (ICAT, iTRAQ, and TMT) or metabolically (SILAC) into amino acids [67–69].

On the other hand, label-free approaches enable relative protein quantitation in complex mixture by (a) measuring the number of acquired MS/MS spectra for all peptides assigned to a given protein or (b) directly comparing the mass spectrometric signal intensity, namely, TIC of MS/MS spectra, assigned to all peptides for a given protein [76–78].

In clinical proteomic applications, the complex nature of human proteome represents a major challenge: in fact, the protein large dynamic range goes from 1–10^5^ or 10^6^ in cells up to 10^9^–10^10^ in plasma [79]. Accordingly, accurate quantitative analysis requires standard operating procedures for handling of specimens and protein sample preparation, increased sensitivity for MS identification, and statistical methods and bioinformatic tools for subsequent interpretation of quantitative proteomic data. Sample preparation is the most important step in order to ensure the reproducibility of proteomic results. As it is well known, no universal protocol exists for the sample preparation although several strategies are adapted according to the type of biological sample [80–83]. In addition to solubilisation procedures of all the proteins in a sample and to removal of contaminants (nucleic acids, polysaccharides, polyphenols, lipids, etc.), the conventional protocols may include depletion of high-abundance proteins from plasma or enrichment processes for urine samples, likely affecting the relative abundance among the components of the protein mixture.

Whatever quantitative proteomic approach is chosen, after protein separation and/or enzymatic digestion, the peptide mixture is injected into a mass spectrometer, usually coupled online with a HPLC. In particular, LC-MS/MS technologies are routinely used for protein/peptide identification in human complex samples [81, 84]. In LC-MS/MS analysis, data are traditionally acquired by Data Dependent Acquisition (DDA) method, whereby only a defined number of the most intense species, observed in the survey MS scan, are selected for fragmentation. Accordingly, this acquisition method leads to a sample underestimation because of the exclusion of low intensity species that remain unidentified [62]. To overcome this issue, Data Independent Acquisition (DIA) methods are now emerging by alternating low and high collision energy or by ion isolation and fragmentation in defined *m*/*z* intervals [85, 86].

As for quantitative proteomics, studies comparing protein levels between two different samples are aimed at detecting differential proteins whose expression significantly changes between conditions (Figure 2). To determine which protein variations are statistically significant a high number of replicates are required and appropriate statistical tests are usually computed for each protein. Such proteomic experiments generally produce complex data, whose interpretation is performed by means of bioinformatic tools available on the net. The whole data, in fact, are analyzed by using specific software in order to (a) cluster the identified proteins according to gene ontology (GO) and/or functional annotation terms; (b) identify relevant biological networks among the identified proteins; and (c) define cellular processes affected by the experimental conditions [87, 88].

### 3.2. Proteomic Application to OAs

Despite the biochemical characterization of OAs, the molecular mechanisms underlying the pathophysiology of these diseases remain poorly understood. However, changes that occur at protein level are now beginning to be explored by using clinical proteomic approaches. The proteome of MMA is so far the most explored thus representing the starting point for proteomic studies applied to OA.

In this context, much of our knowledge arise from (a) ex vivo studies with fibroblasts from MMA patients [89, 90]; (b) in vivo characterization of patients lymphocytes [91]; and (c) more recently analysis of MMA patient' livers [92] (Table 4).

To date, few efforts were dedicated to the finding of molecular signatures and altered cellular pathways, and, as a consequence, to the identification of useful protein targets for designing alternative therapies and/or predicting therapeutic outcomes. A major challenge of clinical proteomic studies related to rare metabolic diseases is often the small sample size due to the unavailability of cells or tissues from patients as well as from age-matched healthy subjects.

To our knowledge, all published studies, related to the proteome of different MMA forms, apply the labeling approach 2D-DIGE coupled to MS/MS techniques.

In this context, Richard et al. reported the first proteomic analysis of patients with isolated MMA by using 2D-DIGE/MS approach, by using MALDI-TOF and MALDI-TOF/TOF for protein identification [89]. Although the small sample size (cultured skin fibroblasts from only two MMA patients), this study represents the first attempt to identify differential expressed mitochondrial proteins in MMA patients with cblH or cblD disorder. Nevertheless, the authors found that proteins related to apoptosis (cytochrome* c*, CYC) and metabolism (succinyl-CoA ligase, SUCLG2) were underexpressed in MMA patients, while oxidative stress proteins (manganese superoxide dismutase, MnSOD) were overexpressed. This could be considered a pioneering study thus leading to hypothesize that a deficit in MUT protein determines a deregulation of cellular and energy metabolism, including the involvement of cytochrome *c* release and ROS overproduction [89].

To partially overcome the small sample size drawback, Hannibal et al. [90] selected three MMACHC patients, whose skin fibroblasts were the only sample available, thus demonstrating that the three related* cblC* cell lines have metabolic and morphological properties suitable to represent a functional model of cobalamin deficiency type C [90]. By using fibroblasts from normal patients as genetically unrelated controls, Hannibal et al. identified protein expression differences, by 2D-DIGE and LC-MS, which were exclusively common to all three* cblC* fibroblasts sources, thus excluding interindividual variability. In agreement with Richard et al. [89], a significant differential expression was observed in* cblC* fibroblasts for proteins related to cellular metabolism and regulation, including cytoskeleton assembly, neurological system, cell signaling, and cellular detoxification [90]. It is noteworthy that no protein expression differences between the normal and* cblC* fibroblasts were observed upon their supplementation with OHCbl, towards which MMACHC patients did not sufficiently respond. Despite the existence of a strict correlation between the* cblC* cell model and MMACHC patient phenotype, this study is limited to patient fibroblasts and not to other cell types, thus lacking a global view of protein variations associated with the metabolic disease.

Interestingly, in a recent study conducted by using 2D-DIGE and LC-MS/MS or MALDI-TOF/TOF, some of the results observed in* cblC* fibroblasts were also confirmed in MMACHC lymphocytes [91]. As for the sample size of this proteomic study, it is noteworthy that the lymphocytes were obtained from 6 MMACHC patients that were compared with six age-matched healthy subjects. Moreover, the enrolled cblC patients were under standard therapy (with OHCbl, betaine, folate, and L-carnitine) showing reduced but still high levels of both plasma tHcy and urine MMA. Due to unavailability of control samples under standard therapy, a limit of this study is that the whole proteomic dataset reflects not only the pathophysiology of the disease, but also the molecular effect inducted by the standard multidrug treatment received by the patients. Nevertheless, in agreement with previous studies on fibroblasts, proteomic analysis of* cblC* lymphocytes showed a deregulation of proteins involved in oxidative stress and cellular detoxification, energy metabolism, cytoskeleton organization, and assembly. However, other proteins, for example, those related to intracellular trafficking and protein folding, were also differentially expressed in* cblC* lymphocytes, thus leading to hypothesize the existence of a tissue-specificity. On the other hand, an underexpression of GSTO1 (glutathione S-transferase omega 1) was observed in* cblC* lymphocytes, as already shown also by Hannibal et al. in fibroblasts [90]. Such an evidence suggests an imbalance of GSH/GSSG metabolism in cblC patients, as previously demonstrated by Pastore et al. in vivo studies [93]. In this context, MS could be a convenient platform for the analysis of protein glutathionylation as PTMs within MMA patients' proteome, thus providing useful information on the disease progression and therapy outcomes, as well as the severity of the oxidative stress to liver and/or kidneys in transplanted patients. In this regard recently a proteomic study was conducted on liver specimens from donors and MMA patients that underwent elective liver or combined liver-kidney transplantation [92]. Once again, a challenge of this study is linked to the availability of healthy donors, here represented by no sex- and age-matched controls, but showing viable and normal hepatocytes. The authors employed 2D-DIGE technology to identify differentially expressed proteins associated to the liver metabolic impairment observed in all six MMA patients diagnosed as vitamin B12 nonresponsive. Accordingly, they found that most of the differentially abundant proteins were involved in metabolic pathways such as energy and carbohydrate metabolism, thus suggesting that a metabolic adaptation occurs to compensate the liver mitochondrial dysfunction, hallmark in MMA. Moreover, the metabolic data, showing the reduction of intermediates of Krebs cycle, provide, together with proteomic results, the first successful attempt to unravel the pathways underlying the hepatic metabolic instability. This integrative approach allowed not only explaining secondary metabolic aspects of MMA (ketonuria and hyperammonemia), but also targeting key enzymes/energy substrates for the design of alternative therapeutic approaches.

To the best of our knowledge, the reported studies clearly show that the proteomic approach is useful for understanding cellular and metabolic processes underlying OA defect. Among MS-based techniques, only the 2D-DIGE analysis platform was utilized to study global protein expression, thus showing its feasibility also to other metabolic diseases. Although it is well known that the 2D-DIGE technique does not detect the whole proteome from a given source in comparison to LC-MS/MS-based methods, the papers here reviewed have successfully used this approach as a starting point to perform differential proteomics on samples derived from patients with IEMs.

## 4. Conclusions

The metabolomic data in OAs, collected to data, derive from extended newborn screening performed using LC-MS/MS platform [94, 95]. Metabolomic analysis of OAs allows quantifying specific biomarkers, which facilitate disease diagnosis, pathogenesis, and therapeutical treatment optimization.

The success of metabolomics in biomarker translation, with respect to other omics techniques, resides in the robustness of the adopted protocols and instrumentations, in the highly quantitative aspect, easily adapted to new assays and already located in many clinical testing laboratories. High automation of technologies represents a great improvement especially when a high number of metabolomic analyses are required. Not only does MS-based newborn screening help in diagnosing or even predicting disease, but also the same techniques can also be used to determine the optimal therapy and monitor or customize the therapeutic dose. However metabolomic strategies need to improve its analytical protocols; indeed they lack a standard operating procedure to analyze biofluids and a proper validation of the obtained results that allow comparing the analytical data between different studies and/or laboratories. A major limit of the metabolomic strategies with respect to other omics techniques, as proteomics, resides in the reduced number of identified metabolites. Compared to proteins, metabolites are a very heterogeneous molecular class due to their different physicochemical properties; so the simultaneous extraction by biofluids is difficult. This reason prevents a good biological interpretation of partial obtained data. The above limit may be overcome by utilizing complementary MS-based technologies. The future challenge in the study of OAs metabolomics is to enable simultaneous targeted and untargeted methods aimed at obtaining sensitive and accurate detection of predetermined metabolites, while allowing detection and identification of still unknown metabolites.

Much of our knowledge of protein changes in OAs arose from proteomic analysis with fibroblasts, lymphocytes, and liver from MMA patients by using 2D-DIGE technology coupled to MS. These clinical proteomic studies are challenged by specimen availability from OA patients as well as healthy subjects. Further investigations, including label-free proteomic approaches, could be employed for relative protein quantitation between specimen from OA patients and healthy controls for their versatility and the required small amount of biological samples.

By a global view of protein variations associated with MMA defect, most of the identified differentially expressed proteins are involved in energy metabolism, cellular detoxification, oxidative stress, and cytoskeleton assembly (Table 4). Despite these first successful results, the molecular mechanisms underlying OA pathophysiology still remain to be deciphered. Therefore, we cannot exclude that increased and/or decreased cellular metabolites could regulate epigenetic modifications and modulate the activities of key enzymes as well as protein PTMs. In this context, MS-based strategies could be a useful platform for the analysis of PTMs from OA patients, thus providing which PTMs impact metabolic processes and cellular functions.

## Supplementary Material

Supplementary Table 1 showing the genetic abnormalities of OAs.

## Figures and Tables

**Table 1:** Multisystem involvement in OAs.

	Hearth	Skeletal muscle	Liver	Pancreas	Kidney	Brain
MSUD	+	+	+	+		++
PA	+	+	+	+		++
MMA	+	+	+	+	+	++
IVA	+	+	+	+		+
GA I	+	+	+	+		+
*β*-KTD	+	+	+	+		+
HMG-CoA lyase D	+	+	+	+		+

**Table 2:** Biomarker in OAs identified in DBS by LC-MS/MS analysis.

MSUD	Val ↑		
	Ile/leu ↑		

PA	C3 ↑	Gly ↑	C3/C0; C3/C4; C3/C16

MMA mut	C3 ↑	Gly ↑	C3/C0; C3/C4; C3/C16

MMA CblA and B	C3 ↑	Gly ↑	C3/C0; C3/C4; C3/C16

MMA CblC and D	C3 ↑	Gly ↑; Met ↑; C16:1OH ↑	C3/C0; C3/C4; C3/C16

IVA	C5 ↑		

GA I	C5DC ↑		C5DC/C4; C5DC/C8; C5DC/C12; C5DC/C3DC

*β*-KTD	C5:1 ↑	C5OHn/↑	

HMG-CoA lyase D	C5OH ↑	C6DCn/↑	

n means normal level, ↑ means increase.

**Table 3:** Organic acids and acylglycines in OAs.

	Urinary organic acids detected by GC-MS	Urinary acylglycines detected by LC-MS/MS
MSUD	2-Keto-isocaproic acid ↑	
	2-OH-isovaleric acid ↑	
	2-Keto-isovaleric acid ↑	
	2-Keto-3-methylvaleric acid ↑	
	2-OH-3-methylvaleric acid ↑	

PA	3-OH-propionic acid ↑	Tiglylglycine ↑
	Methylcitric acid ↑	Propionylglycine ↑

MMA	Methylmalonic acid ↑	
	Methylcitric acid ↑	

IVA	3-Hydroxyisovaleric acid ↑	Isovalerylglycine ↑

GA I	Glutaric acid ↑	Glutarylglycine ↑
	3-OH glutaric acid ↑	

*β*-KTD	2-Methyl-3-OH butyric acid ↑	Tiglylglycine ↑
	2-Methyl-acetoacetic acid ↑	

HMG-CoA lyase D	3-Methyl-glutaconic acid ↑	

↑ means increase.

**Table 4:** Summary of proteomic results from specimens of MMACHC patients.

Cellular system	Main results	MS-based proteomic technology	References
Fibroblasts	Underexpression of proteins related to apoptosis and metabolism. Overexpression of oxidative stress proteins	2D-DIGE/MALDI-TOF and MALDI-TOF/TOF	Ebhardt et al., 2006 [87]

Fibroblasts	Differentially expressed proteins related to cellular metabolism and regulation, cytoskeleton assembly, neurological system, cell signaling, and detoxification	2D-DIGE/LC-MS	Richard et al., 2011 [89]

Lymphocytes	Deregulation of proteins involved in oxidative stress and cellular detoxification, energy metabolism, cytoskeleton organization, and assembly	2D-DIGE/LC-MS/MS and MALDI-TOF/TOF	Hannibal et al., 2015 [90]

Liver	Differentially expressed proteins involved in energy and carbohydrate metabolism	2D-DIGE/LC-MS/MS and MALDI-TOF/TOF	Caterino et al., 2015 [91]

## Abbreviations

2D-DIGE: Two-Dimensional Differential In-Gel Electrophoresis
Cbl: Cobalamin
C0: Free Carnitine
C3: Propionylcarnitine
C3DC: Malonyl-L-carnitine
C4: Butyryl-L-carnitine
C5: Valeryl-L-carnitine
C5:1: Tiglyl-L-carnitine
C5DC: Glutaryl-L-carnitine
C5OH: Hydroxyglutaryl-L-carnitine
C8: Octanoyl-L-carnitine
C12: Dodecanoyl-L-carnitine
C16: Hexadecanoyl-L-carnitine
DBS: Dried blood spot
DDA: Data Dependent Acquisition
DIA: Data Independent Acquisition
GA I: Glutaric acidemia type I
GC: Gas chromatography
HMG-CoA: 3-Hydroxy-3-methylglutaryl-CoA
HR-MS: High-resolution mass spectrometry
ICAT: Isotope Coded Affinity Tags
IEMs: Inborn errors of metabolism
iTRAQ: Isobaric Tag for Relative and Absolute Quantitation
IVA: Isovaleric acidemia
LC: Liquid chromatography
MMA: Methylmalonic acidemia
MS: Mass spectrometry
MSMS: Tandem mass spectrometry
MSUD: Maple syrup urine disease
MUT: Methylmalonyl-CoA mutase
NMR: Nuclear magnetic resonance
OAs: Organic acidurias
PA: Propionic acidemia
PCA: Principal components analysis
PCCA: Propionyl-CoA carboxylase alpha genes
PCCB: Propionyl-CoA carboxylase beta genes
PLS: Partial least squares
PTMs: Posttranslational modifications
SILAC: Stable Isotope Labeling by Amino acids in Cell Culture
SpC: Spectral Counting
TIC: Total Ion Current
TMT: Tandem Mass Tags
*β*-KTD: Beta ketothiolase deficiency.
