# Supplementary material for: Mass Spectrometry-Based Metabolomic and Proteomic Strategies in Organic Acidemias
Source: Biomed Res Int. 2016 Jun 14;2016:9210408. doi: 10.1155/2016/9210408 (PMC4923558; doi:10.1155/2016/9210408)
Supplement: Supplementary file 1 — Supplementary Table 1 showing the genetic abnormalities of OAs. [file 9210408.f1.pdf]

Supporting information for

Mass spectrometry-based metabolomic and proteomic strategies in organic acidemias.

Imperlini Esther[1], Santorelli Lucia[2], Orrù Stefania[2,3], Scolamiero Emanuela[2], Ruoppolo Margherita[2,4], Caterino Marianna[4]*

1) Fondazione IRCCS SDN, Naples, Italy
2) CEINGE Biotecnologie Avanzate scarl, Naples, Italy
3) Dipartimento di Scienze Motorie e del Benessere, Università di Napoli "Parthenope", Naples, Italy
4) Dipartimento di Medicina Molecolare e Biotecnologie Mediche, Universita` degli Studi di Napoli ‘‘Federico II’’, Naples, Italy

Supplementary Table 1. Genetic abnormalities of OAs.

OAs	Gene	Mutations*	References
MSUD	DBT	DBT, 124-BP DEL	Herring et al. (1991)
		DBT, PHE215CYS	Fisher et al. (1991)
		DBT, 17-BP INS	Chuang et al. (1991)
		DBT, 78-BP DEL	Mitsubuchi et al. (1991)
		DBT, 126-BP INS	Tsuruta et al. (1998)
		DBT, TER422LEU	Tsuruta et al. (1998)
		DBT, ILE37MET	Tsuruta et al. (1998)
		DBT, GLY323SER	Tsuruta et al. (1998)
		DBT, 2-BP DEL, 88AT	Fisher et al. (1993)
		DBT, 4.7-KB DEL	Chi et al. (2003)
		DBT, HIS391ARG	Chuang et al. (2004)
		DBT, SER133TER	Chuang et al. (2004)
	BCKDHB	BCKDHB, 11-BP DEL	Nobukuni et al. (1991)
		BCKDHB, ARG183PRO	Edelmann et al. (2001)
		BCKDHB, HIS156TYR	Chuang et al. (2004)
		BCKDHB, VAL69GLY	Chuang et al. (2004)
		BCKDHB, 4-BP DEL, IVS9	Chuang et al. (2004)
		BCKDHB, 8-BP INS, NT1109	Chuang et al. (2004)
	BCKDHA	BCKDHA, TYR393ASN	Zhang et al. (1991); Chuang et al. (1994); Matsuda et al. (1990); Dariush et al. (1991); Fisher et al. (1991); Mitsubuchi et al. (1992); Wynn et al. (1998); Puffenberger (2003)
		BCKDHA, 8-BP DEL, 887-894	Zhang et al. (1989); Chuang et al. (1994)
		BCKDHA, GLY245ARG	Chuang et al. (1995); Wynn et al. (1998);
		BCKDHA, PHE364CYS	Chuang et al. (1995); Wynn et al. (1998);
		BCKDHA, ARG220TRP	Wynn et al. (1998)
		BCKDHA, GLY204SER	Wynn et al. (1998)
		BCKDHA, THR265ARG	Wynn et al. (1998)
		BCKDHA, CYS219TRP	Chuang et al. (2004)

		BCKDHA, 1-BP DEL, 117C	Quental et al. (2008); Quental et al. (2009)
PA	PCCB	PCCB, ARG412TRP	Tahara et al. (1990); Ugarte et al. (1999)
		PCCB, 8-BP DEL, NT3	Ohura et al. (1993)
		PCCB, 12-BP INS, 14-BP DEL, NT1218	Rodriguez-Pombo et al. (1998)
		PCCB, 1-BP INS, 1170T	Rodriguez-Pombo et al. (1998)
		PCCB, GLU168LYS	Rodriguez-Pombo et al. (1998)
		PCCB, THR428ILE	Ohura et al. (1993)
		PCCB, 3-BP INS, 1540CCC	Ravn et al. (2000)
		PCCB, TYR435CYS	Yorifuji et al. (2002)
		PCCB, IVS6, A-G, +462	Rincon et al. (2007)
	PCCA	PCCA, 4-BP DEL, 1824IVS, +3	Clavero et al. (2004); Richard et al. (1997)
		PCCA, 9-BP DEL, 1771IVS, -2	Richard et al. (1997)
		PCCA, 2-BP INS, 1824IVS, +3	Richard et al. (1997)
		PCCA, ARG288TER	Campeau et al. (1999)
		PCCA, MET348LYS	Richard et al. (1999)
		PCCA, IVS14, A-G, -1416	Rincon et al. (2007)
MMA	MUT	MUT, GLN17TER	Fenton et al. (1987); Ledley et al. (1990)
		MUT, TRP105ARG	Jansen and Ledley (1990)
		MUT, ALA378GLU	Jansen and Ledley (1990)
		MUT, ARG93HIS	Raff et al. (1991); Ledley and Rosenblatt (1997)
		MUT, GLY717VAL	Ledley et al. (1990) ; Crane et al. (1992); Worgan et al. (2006)
		MUT, GLU117TER	Ogasawara et al. (1994)
		MUT, 2-BP DEL, 769CA	Ogasawara et al. (1994)
		MUT, GLY623ARG	Qureshi et al. (1994)
		MUT, GLY703ARG	Qureshi et al. (1994)
		MUT, ASN219TYR	Acquaviva et al. (2001); Berger et al. (2001)
		MUT, ARG108CYS	Worgan et al. (2006)
		MUT, GLY215SER	Cavicchi et al. (2006)
		MUT, IVS11, C-A, -891	Rincon et al. (2007); Martinez et al. (2005)

	MUT, 1808G-A	Rincon et al. (2007
MMACHC	MMACHC, 1-BP DUP, 271A	Lerner-Ellis et al. (2006)
	MMACHC, LEU116PRO	Lerner-Ellis et al. (2006)
	MMACHC, ARG132TER	Lerner-Ellis et al. (2006); Morel et al. (2006); Ben-Omran et al. (2007); Lerner-Ellis et al. (2009)
	MMACHC, ARG111TER	Morel et al. (2006); Lerner-Ellis et al. (2009)
	MMACHC, ARG161GLN	Bodamer et al. (2001); Morel et al. (2006); Tsai et al. (2007); Lerner-Ellis et al. (2009); Liu et al. (2010)
	MMACHC, TRP203TER	Liu et al. (2010)
	MMACHC, 3-BP DEL, 658AAG	Liu et al. (2010)
	MMACHC, GLU92ASP	Komhoff et al. (2013)
	MMACHC, GLU92GLU	Komhoff et al. (2013)
	MMACHC, GLY155GLU	Komhoff et al. (2013)
MMAA	MMAA, 4-BP DEL, 592ACTG	Dobson et al. (2002)
	MMAA, 8-BP INS, NT260	Dobson et al. (2002)
	MMAA, GLN95TER	Dobson et al. (2002)
	MMAA, TYR207CYS	Dobson et al. (2002)
	MMAA, ARG145TER	Lerner-Ellis et al. (2004)
MMAB	MMAB, ARG186TRP	Dobson et al. (2002)
	MMAB, IVS3, G-A, -1	Dobson et al. (2002)
	MMAB, 5-BP DEL, NT572	Dobson et al. (2002)
	MMAB, ILE96THR	Jorge-Finnigan et al. (2010)
	MMAB, SER174FS	Brasil et al. (2015)
	MMAB, ARG191TRP	Jorge-Finnigan et al. (2010)
	MMAB, IVS4AS, G-C, -1	Jorge-Finnigan et al. (2010)
	MMAB, 290G-A	Jorge-Finnigan et al. (2010)
	MMAB, HIS183LEU	Brasil et al. (2015)
	MMAB, 3-BP DUP, NT568	Brasil et al. (2015)
MMADHC	MMADHC, LEU259PRO	Coelho et al. (2008); Suormala et al. (2004)
	MMADHC, THR182ASN	Coelho et al. (2008)

		MMADHC, TYR249CYS	Coelho et al. (2008)
		MMADHC, 8-BP DEL, NT57	Coelho et al. (2008); Suormala et al. (2004)
		MMADHC, ARG54TER	Coelho et al. (2008)
		MMADHC, 18-BP DUP, NT307	Coelho et al. (2008)
		MMADHC, ARG250TER	Goodman et al. (1970); Coelho et al. (2008)
		MMADHC, 1-BP DUP, 419A	Coelho et al. (2008)
		MMADHC, IVS7DS, 4-BP DEL	Coelho et al. (2008)
	LMBRD1	LMBRD1, 1-BP DEL, 1056G	Rutsch et al. (2009)
		LMBRD1, 2-BP DEL, 515AC	Rutsch et al. (2009)
		LMRBD1, 1-BP DEL, 404C	Rutsch et al. (2009)
	HCFC1	HCFC1, 455A-G	Gedeon et al. (1991); Huang et al. (2012)
		HCFC1, SER225ASN	Huang et al. (2012)
		HCFC1, ALA115VAL	Yu et al. (2013)
		HCFC1, ALA73VAL	Yu et al. (2013)
		HCFC1, ALA73THR	Yu et al. (2013)
IVA	IVD	IVD, LEU13PRO	Vockley et al. (1991)
		IVD, GLY170VAL	Vockley et al. (1991)
		IVD, 1-BP DEL, 1177T	Vockley et al. (1991)
		IVD, 90-BP DEL, NT145	Vockley et al. (1992)
		IVD, IVS7AS, G-A, -1	Vockley et al. (2000)
		IVD, ARG21CYS	Vockley et al. (2000)
		IVD, ALA282VAL	Ensenauer et al. (2004)
GA I	GCDH	GCDH, TYR295HIS	Biery and Goodman (1992); Goodman et al. (1995)
		GCDH, ALA421VAL	Biery et al. (1996)
		GCDH, THR416ILE	Anikster et al. (1996)
		GCDH, ARG402TRP	Zschocke et al. (2000)
		GCDH, GLU365LYS	Kolker et al. (2001); Zschocke et al. (2000)
		GCDH, IVS1, G-T, +5	Greenberg et al. (1995)
		GCDH, ALA293THR	Biery et al. (1996)

		GCDH, VAL400MET	Biery et al. (1996); Marti-Masso et al. (2012)
		GCDH, ARG227PRO	Biery et al. (1996)
β-KTD	ACAT1	ACAT1, ALA347THR	Fukao et al. (1991)
		ACAT1, GLY150ARG	Fukao et al. (1991); Schutgens et al. (1982)
		ACAT1, IVS8, G-T, +1	Fukao et al. (1991)
		ACAT1, IVS10, A-C, -2	Fukao et al. (1991)
		ACAT1, IVS10, G-C, -1	Fukao et al. (1991)
		ACAT1, IVS11, T-C, +2	Daum et al. (1973), Fukao et al. (1993)
		ACAT1, MET1LYS	Daum et al. (1973), Fukao et al. (1993)
		ACAT1, GLY379VAL	Fukao et al. (1994); Steingrimsdottir et al. (1992)
		ACAT1, GLN272TER	Fukao et al. (1994)
		ACAT1, 3-BP DEL, 1033GAA	Sewell et al. (1998)
		ACAT1, 1-BP INS, 1083A	Sewell et al. (1998)
		ACAT1, ASN93SER	Fukao et al. (1998)
		ACAT1, ILE312THR	Fukao et al. (1998)
		ACAT1, ALA333PRO	Fukao et al. (1998)
		ACAT1, GLN145GLU	Fukao et al. (2002)
		ACAT1, 1-BP DEL, 149C	Fukao et al. (1998)
HMG-CoA Lyase D	HMGCL	HMGCL, 2-BP DEL, NT202	Mitchell et al. (1992), Mitchell et al. (1993)
		HMGCL, VAL70LEU	Mitchell et al. (1992)
		HMGCL, 930-BP DEL, EX3-6DEL	Wang et al. (1996)
		HMGCL, ARG41GLN	Mitchell et al. (1998)
		HMGCL, GLU279LYS	Muroi et al. (2000)
		HMGCL, 2-BP DEL, TT, CODON 305	Mitchell et al. (1998)

* Mutations, cataloged in OMIM, are selected by criteria for inclusion as described by Amberger et al. Nucleic Acids Research 43:D789, 2015.
